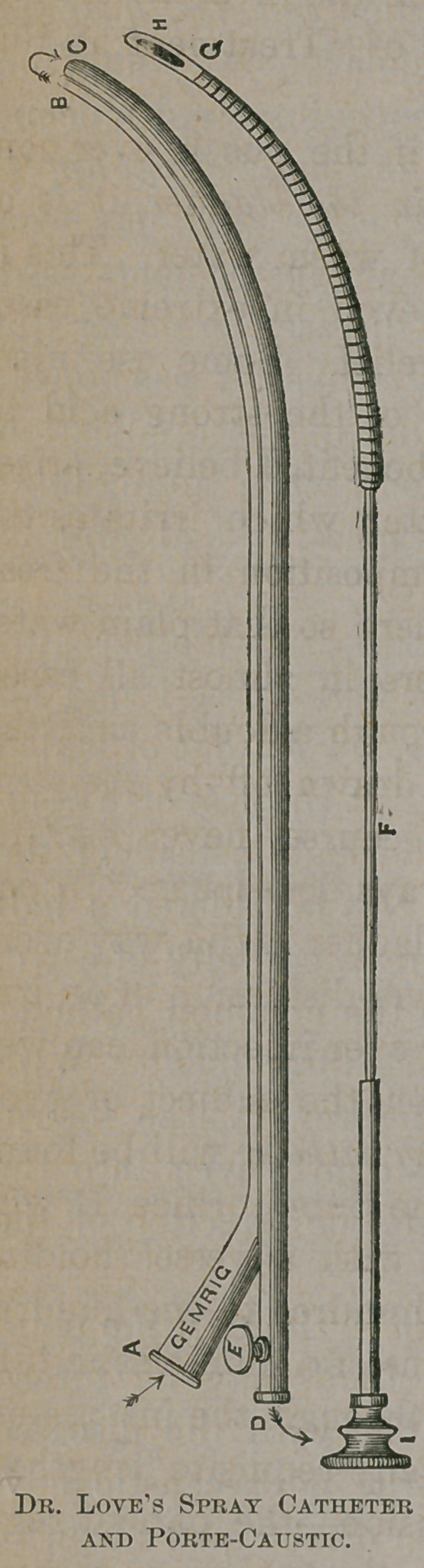# A New Instrument for Treating Locally Certain Diseases of the Genito-Urinary Organs, Male or Female

**Published:** 1871-12

**Authors:** Wm. Abram Love

**Affiliations:** Atlanta, Georgia


### A NEW INSTRUMENT

### *For Treating Locally Certain Diseases of the Genito-Urinary Passages, Male or Female.*

BY WM. ABRAM LOVE, M.D., ATLANTA, GEORGIA.

That there is an evident tendency on the part of the profession—or, at least, the leading members who are devoting their time and talent to the subject—to the abandonment of the so-called *specifics* in the treatment of inflammations (specific or non-specific) of the male genito-urinary passages, and to resort to the direct application of remedial agents, is evidenced in the more recent writings of such men as Acton, Lee, Durkee, Cullevier, not to mention the many who have, of late years, added so much to our knowledge in the local treatment of diseases of females—Bumstead, Beıkely Hill, and others. Niemeyer, too, adds his testimony in favor of *injections* as speedily arresting blenorrhagia in the first stage, and does not resort to "cubebs and copaiba," even in the third stage, until injections have failed. He furthermore states, that "the fact that their action is less certain in this stage is because, when of long standing, the inflammation is no longer confined to the more accessible anterior portions of the urethra, but has spread into the posterior regions, where an injection can reach it less easily."

The specific virus of gonorrhœa—though usually, in the outset, confined to a narrow limit near the external meatus—soon spreads itself along the urethra to the prostate, or even to the internal surface of the bladder. Under such circumstances, the parts reached by injections may be remedied, while the deeper-seated organs, not so reached, are preyed upon by the pus germs. "Pus," says Lionel S. Beale, (Kidney Diseases and Urinary Deposits, p. 369–370), "grows the faster the more freely nutrient matter is supplied to it, and it lives upon the pabulum which is really required for the nutrition of healthy tissue. *The pus lives faster than any healthy tissue.*" In this way, the healthy pabulum at some point along the line is consumed, the tissue wasted, and a deeperseated disease fixed in the character of an ulcer, or chronic circumscribed suppuration or inflammation, involving the mucous or submucous tissues. An injection *sufficiently strong* to arrest this *pus growth* at this particular point would be injurious to the healthy, or young recently-impaired mucous membrane. To overcome this obstacle, *and make direct application to the seat of the disease, and to no other part*, has been a somewhat difficult task, with the ordinary instruments used for the local treatment of this class of diseases, in either the male or female subject. Lallemand's instrument, it was hoped, would overcome the difficulty; and it did, in the application of the solids or powders, to some extent, when admissible; but they are objectionable in many instances, and absolutely injurious in others.

To overcome this, and at the same time serve other purposes—allusion to which will be made hereafter—I had an instrument constructed some years since, a wood cut of which is herewith presented. (See page 508.) It consists of a common male catheter (size 6), open at the end instead of the sides. On the inner or curved side of this is soldered a half catheter of the same metal (silver), giving a capacity of a No. 2 catheter between the two. Through the common catheter passes a porte-caustic, as in Lallemand's instrument. With this in place, and *fixed* by the thumb-screw E, the instrument is ready for introduction to any depth, even into the bladder. The arrows in the cut indicate the direction of the fluid used.

Fixing a common-gauged hypodermic syringe in the point A, any given number of minims of fluid may be forced through and out at B, to come in contact with the diseased surface, and return through C to D—the porte-caustic having been removed after the introduction of the instrument to the requisite depth. If it is desired to throw in or through a larger quantity of fluid, a small piece of india rubber tubing may connect the orifice A with a Davidson's or other syringe or apparatus for washing out the urethra, the bladder, or uterine cavity, where that organ will tolerate the wash. Where it is desired to use the solid or powdered nitrate of silver, sulphate zinc, or other substances, the instrument may be used as Lallemand's, placing the substance in the cavity at G, with this additional advantage: Filling the cavity G with nitrate of silver or other soluble substance, inserting the instrument to the proper depth, and pushing through the porte-caustic into the urethral canal, water or any other solvent may be thrown in through the side tube A B by the hypodermic syringe, and the solution made at the diseased point. Then the porte-caustic may be removed and the cauterized surface washed off, or any other application made without changing the instrument in the urethral canal, or the uterine cavity.

Dr. Love's Spray Catheter and Porte-Caustic.

For cleansing the intra-cervical canal, or the uterine cavity, I have found the instrument very convenient. At the point H in the porte-caustic, there is a small eye passing through. Using this to fasten to the porte-caustic soft floss thread, (making a mop somewhat after the fashion of a bristle probang, for removing foreign bodies from the œsophagus), I have succeeded in cleansing these cavities of mucous pus, etc., effectually, by turning the porte-staff on its own axis. Then the syringe may be used, and medicated fluids applied as to the urethral surface. In this way, tents of cotton or lint floss may be saturated *in utero*, or *within the cervical canal;* whereas, if they are saturated first, and then the effort made to introduce them, the fluids are pressed out before they reach their destination. For intra-uterine medication, the instrument should not be so much curved, and may be of larger caliber. For the female bladder, it should be constructed nearer like the female catheter.

In irritability or catarrh of the bladder, or suppuration in that organ—whether due to gonorrhœa, gout, stricture, cold, or inability to entirely empty that viscus, the instrument will be found invaluable. Dr. L. S. Beale, in the invaluable work referred to above, says, under the head of "Treatment of Pus in the Urine," (p. 369):

"In all bad cases, more especially if the pus is ever converted into a ropy, mucous-like mass, *in the bladder*, it is of the first importance to use injections of warm water. This is a very simple operation, and affords, even in extreme cases which cannot be cured, the greatest relief. Some use injections of diluted nitric acid, (one drop of the strong acid to each ounce of water); but the chief benefit, I believe, arises from removing the decomposing matter, which irritates the mucous membrane and excites decomposition in the fresh urine as fast as it reaches the bladder; so that plain water (warm distilled or rain water) answers, in almost all cases, perfectly well. It may be injected through a double catheter, or through an ordinary catheter, and drawn off by the same instrument. The bladder should, of course, never be fully injected, as distension of its coats always does harm. In bad cases, it is necessasy to wash out the bladder in this way every day. Those who have suffered from over-distension of an irritable bladder, from either retention or over-injection, can well appreciate the caution of Dr. Beale on the subject of over-injection. It is in such cases this *spray catheter* will be found peculiarly applicable. Connecting the outer orifice B with the syringe—or, what is preferable, with a vessel holding water at the proper temperature (one hundred to one hundred and ten degrees Fahrenheit)—by means of a gum elastic tube as a siphon, the fluid may be poured through the instrument and bladder in any quantity, or for any requisite length of time, without the risk of over-distension—the patient regulating the *inward* flow by pressure on the tubing, or the *outward* flow with the point of the finger or pressure on another piece of tubing, which may be connected with the outer end D, and serve to convey the fluid into some proper vessel. In this way the bladder may be distended at pleasure, its walls placed under any given pressure, or medicated fluids thrown through at will.

I well remember two cases—one a female, the other a male—where paralysis of the bladder had followed over-distension from retention of urine, rendering the repeated and long-continued use of the catheter necessary. In both the urine was alkaline, fetid, and filled with pus mucous corpuscles, and bladder epithelial from floor and fundus. After nearly every other plan of treatment had failed, the douche, or thorough washing out of the organ as described above—at first twice a day, afterward at longer intervals—with iron, quassia, quinia, nux vomica, etc., by the stomach, brought speedy relief and restoration to a healthy condition, local as well as constitutional. If solvents of urinary calculi can accomplish anything, they may be in this way resorted to with least trouble to patient or practitioner.

Most of the double catheters in use have the disadvantage of becoming clogged; and, to some extent, the same objection will apply to this, or to any instrument of like caliber. This instrument, in this respect, has the advantage of having the *internal orifice* accessible by the porte-caustic, by which the obstructions may be easily removed. Another and great advantage in it is, that *any amount of surface*, from a mere line to the whole length of a cavity or canal, *may be medicated with fluids or solids, and, with a little practice, the application kept within the desired limits, the parts above and below escaping the application.* There is certainly no necessity for burning out or cauterizing the whole urethral passage, simply to reach a small circumscribed ulcer. Such has been the practice, however, in many instances, in *chronic gonorrhœa*, *gleet*, *stricture*, etc., in the male, to say nothing of the *wholesale* applications in circumscribed endo-cervicitis and endometritis, or "ulceration of the womb," in the female.

In *spasmodic stricture*, warm water—or, preferably, warm aqueous infusions of belladonna, opium, etc.—brought in contact with the seat of the stricture, will often allay the spasm and admit of the passage of an instrument, when other means have failed. The warmth, as well as the medicinal agents, are brought *directly in contact with the affected part;* as the stricture gives way, the application is continued further up the passage, until the bladder is entered and the patient relieved.

In cases of over-engorgement of the prostate, a cold douche to the parts (and to the rectum) will, in some instances, serve to remove the obstacle in the way of the passage of a catheter. I have not tried connecting an *atomizer* with the instrument for local application of remedial agents, but do not doubt the practicability of the operation, or the benefits to be derived from such treatment in certain cases.

I have not found *orchitis* to follow the use of the instrument, as it does the use of injections in very many instances, particularly where the solutions used by injection are very strong or the temperature too low. I have attributed this to the fact, that when the seat and extent of the disease along the urethra is ascertained by exploration with the globe or olive-pointed sound, which is generally satisfactory, the application can be confined within proper bounds, the remaining parts saved from undue medication or interference with its normal action, and the irritation does not extend itself to the testicle.

This off-hand communication was not commenced with any intention of entering into the pathology or treatment of any of the diseases herein referred to, but simply for the purpose of calling the attention of such of my professional brethren as may be interested in the subject, to the advantages of this form of instrument, the use of which has saved me much annoyance, and proved of incalculable benefit to some of my patients, *particularly those who have suffered from disease of the bladder.*

---

Diagnostication of Bright's Disease.—At a recent meeting of the Suffolk District Medical Society (Boston Medical and Surgical Journal), Dr. H. W. Williams referred to four cases, occurring lately, in which he had diagnosticated Bright's disease of the kidneys by the characteristic changes in the retina, as discovered by the ophthalmoscope. Dr. Williams did not regard the degeneration of the retina as especially belonging to the early stages of the disease, yet it was not infrequently the first symptom discovered.

## Figures and Tables

**Figure f1:**